# The Protective Mechanism of Deuterated Linoleic Acid Involves the Activation of the Ca^2+^ Signaling System of Astrocytes in Ischemia In Vitro

**DOI:** 10.3390/ijms222413216

**Published:** 2021-12-08

**Authors:** Egor A. Turovsky, Elena G. Varlamova, Sergey V. Gudkov, Egor Y. Plotnikov

**Affiliations:** 1 Federal Research Center “Pushchino Scientific Center for Biological Research of the Russian Academy of Sciences”, Institute of Cell Biophysics of the Russian Academy of Sciences, 142290 Pushchino, Russia; 2 Prokhorov General Physics Institute of the Russian Academy of Sciences, 38 Vavilove St., 119991 Moscow, Russia; s_makariy@rambler.ru; 3 A.N. Belozersky Institute of Physico-Chemical Biology, Lomonosov Moscow State University, 119992 Moscow, Russia; plotnikov@belozersky.msu.ru; 4 V.I. Kulakov National Medical Research Center of Obstetrics, Gynecology and Perinatology, 117997 Moscow, Russia

**Keywords:** oxygen-glucose deprivation, cell death, cortex, astrocyte, cell protection, signaling, ROS, deuterated linoleic polyunsaturated fatty acid

## Abstract

Ischemia-like (oxygen-glucose deprivation, OGD) conditions followed by reoxygenation (OGD/R) cause massive death of cerebral cortex cells in culture as a result of the induction of necrosis and apoptosis. Cell death occurs as a result of an OGD-induced increase in Ca^2+^ ions in the cytosol of neurons and astrocytes, an increase in the expression of genes encoding proapoptotic and inflammatory genes with suppression of protective genes. The deuterated form of linoleic polyunsaturated fatty acid (D4-Lnn) completely inhibits necrosis and greatly reduces apoptotic cell death with an increase in the concentration of fatty acid in the medium. It was shown for the first time that D4-Lnn, through the activation of the phosphoinositide calcium system of astrocytes, causes their reactivation, which correlates with the general cytoprotective effect on the cortical neurons and astrocytes in vitro. The mechanism of the cytoprotective action of D4-Lnn involves the inhibition of the OGD-induced calcium ions, increase in the cytosolic and reactive oxygen species (ROS) overproduction, the enhancement of the expression of protective genes, and the suppression of damaging proteins.

## 1. Introduction

Cerebral ischemia, which occurs for many reasons, leads to a cascade of intracellular events leading to necrotic or apoptotic cell death. Such events include a decrease in the level of oxygen and glucose entering cells, a global increase in the concentration of Ca^2+^ ions in the cytosol ([Ca^2+^]_i_), an increased release of excitatory neurotransmitters by cells into the extracellular environment, and, especially, multiple increases in reactive oxygen species (ROS) production [1,2]. Many neuroprotective drugs have not been shown to be highly effective in preventing or recovering from a stroke, since these drugs poorly penetrate the blood–brain barrier.

The brain is the second organ after adipose tissue that contains the most lipids—more than 50% of the total dry weight of the brain. Lipids are structural components of nerve cell membranes. Brain lipids include 50% phospholipids, 40% glycolipids, 10% cholesterol, and cholesterol ester and traces of triglycerides. Polyunsaturated fatty acids (PUFAs) account for 25–30% of the total fatty acids (FAs) in the human brain and include docosahexaenoic acid and arachidonic acid, which play an important signaling role in health and various brain pathologies [3]. Since the brain tissue is very rich in lipids, disturbances in lipid homeostasis lead to a number of neurodegenerative diseases—Alzheimer’s, Parkinson’s, and others [4]. In stroke, ischemia/reperfusion is accompanied by a sharp accumulation of PUFAs and their derivatives, which can trigger mechanisms of damage to brain cells [5,6]. In addition, during hypoxia and ischemia, lipid peroxidation increases due to the overproduction of ROS [7,8].

The search for effective methods of therapy and the study of neuroprotective mechanisms are urgent tasks of research in the field of neurobiology, including the activation of cell defense systems using exogenous PUFAs. Lipid peroxidation (LPO) results in the elimination of a hydrogen atom from the methylene group under the action of ROS. Natural PUFAs such as linoleic acid undergo autooxidation of ROS, resulting in the formation of toxic, reactive carbonyl forms that lead to DNA damage and inflammation. However, deuterated PUFAs (D-PUFAs), such as deuterated linoleic acid, stop this self-oxidation and therefore protect against oxidative stress. Deuteration of PUFAs is the process of selectively replacing a hydrogen atom with a more stable hydrogen isotope, deuterium, resulting in stronger hydrogen bonds in a fatty acid molecule. This method of changing the structure of PUFAs has already shown neuroprotective properties in neurodegenerative diseases, slowing down LPO under the action of ROS [9]. Thus, the therapeutic effect of D-PUFAs in models of Parkinson’s disease was in the form of a decrease in the LPO level, restoration of the mitochondria membrane potential in PLA2G6 (phospholipaseA2 group VI) mutant human fibroblasts, and the partial rescuing of the locomotor abnormalities in iPLA2-VIAnull [10]. Exogenous administration of D-PUFAs into primary neuron–glia co-cultures from rat cortex exhibited efficacy against oligomeric α-syn-mediated cell death and LPO [11]. It has been shown that chronic supplementation with D-PUFAs, especially 0.8% D-linoleic (D-Lnn), had significant beneficial effects on Parkinson’s disease against α-syn-induced motor deficits, synaptic pathology, oxidative damage, mitochondrial dysfunction, etc., [12]. These data support the clinical evaluation of D-PUFA as a neuroprotective agent for the treatment of PD.

Linoleic and α-linoleic acids are precursors of long-chain PUFAs and must be obtained from food, since mammals cannot synthesize these acids de novo. Despite the available evidence of the neuroprotective effect of D4-Lnn, the molecular mechanisms of the regulation of these processes are still poorly understood; therefore, the aim of this work was to study the neuroprotective effect of D4-Linoleic acid (D4-Lnn), which contains four deuterium atoms at the 9, 10, 12, and 13 positions.

## 2. Results

### 2.1. Effect of Different Concentrations of D4-Lnn on the Induction of Necrosis and Apoptosis in Cerebral Cortex Cells after Ischemia/Reoxygenation

**Figure 1:**
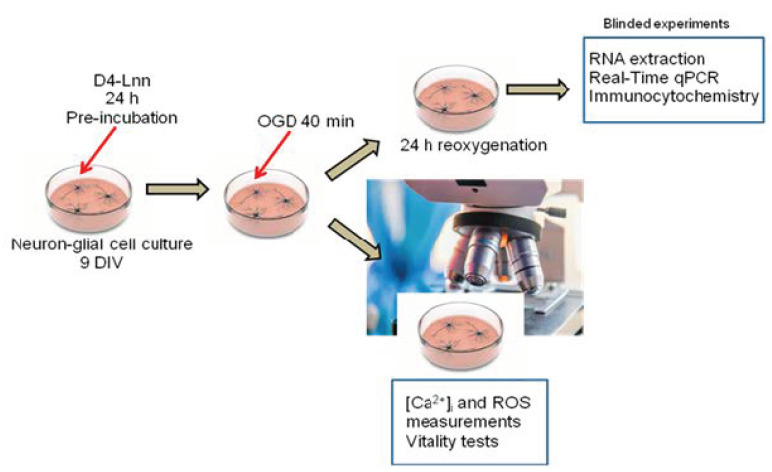
Scheme of the study of the mechanisms of the cytoprotective action of D4-Lnn in the OGD and OGD/R conditions. Cells were grown up to day 9 in vitro and D4-Lnn or its non-deuterated form was added for 24 h. Then, the cells were loaded with fluorescent probes, and Ca^2+^ dynamics in the cytosol, ROS production, and vitality tests were performed during OGD. Part of the cells after 40 min OGD was returned to the CO_2_ incubator for 24 h, which corresponded to the conditions of reoxygenation. After OGD/R, total RNA was isolated from part of the cells and PCR analysis was performed, and part of the culture was used for immunocytochemical staining.

The studies were carried out according to the scheme shown in Figure 1. Primary neuroglial cultures of the mouse cerebral cortex were grown for up to 9 days in a CO_2_ incubator, after which D4-Lnn or its non-deuterated form (Lnn) was supplemented and used for experiments.

**Figure 2:**
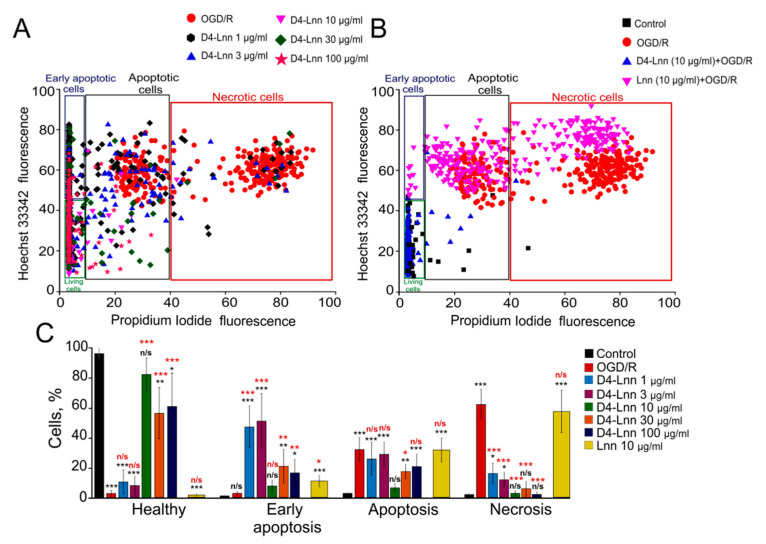
The effect of different concentrations of D4-Lnn on induction of apoptosis and necrosis in the cortical cells after 40 min OGD and 24 h reoxygenation: (**A**) Cytogram demonstrating the viability of cortical cells after OGD/R without pre-incubation with D4-Lnn and after OGD/R with 24 h pre-incubation with different concentrations of D4-Lnn. (**B**) Comparison of the effect of 10 µg/mL D4-Lnn and Lnn on cell survival after 24 h of OGD/R. Control-cells were not exposed to OGD/R; OGD/R: cells of the cerebral cortex after 24 h OGD/R without pre-incubation with PUFAs; X-axis: the intensity of PI fluorescence; Y-axis: the intensity of Hoechst 33342 fluorescence. Cells were stained with the probes after 24 h the OGD/R. Panels A and B show cells (several hundred) in one experiment. (**C**) Effects of different D4-Lnn concentrations and 10 µM Lnn on the induction of necrosis and apoptosis after 24 h OGD/R. The percentage of healthy cells and cells with early apoptosis, apoptosis, and necrosis. Statistical significance was assessed using one-way ANOVA followed by the Tukey–Kramer test. Comparison of experimental groups relative to control: n/s—data not significant (*p* > 0.05), * *p* < 0.05, ** *p* < 0.01 and *** *p* < 0.001. Statistical differences indicated by red asterisks represent comparison of treatment groups relative to OGD/R. Panel C shows the average results obtained from four cell cultures. N (number of animals used for cell cultures preparation) = 4.

To study the neuroprotective effect of D4-Lnn on the mouse cerebral cortex cells, we selected the following concentration range: 1, 3, 10, 30, and 100 µg/mL. It was found that, in the absence of any effect in neuroglial cultures on the 10 th day, practically no cytotoxic effect of D4-Lnn was recorded (no more than 2–3% of cells) (Figure 1, control). Ischemia-like conditions (oxygen-glucose deprivation, OGD) lasting 40 min, alternating with reoxygenation in a CO_2_ incubator for 24 h, led mainly to the activation of late stages of apoptosis (42 ± 13% of cells) and necrotic processes in 62 ± 21% of cells (Figure 2A–C-OGD/R, Supplementary, Figure S1—OGD/R). To study the protective effect of D4-Lnn against the cytotoxic effect of ischemia/reoxygenation, study cells were first incubated with various concentrations of D4-Lnn for 24 h, after which the OGD/R conditions were created. Incubation of cortical cells with 1 or 3 µg/mL D4-Lnn resulted in a decrease in the number of necrotic cells by more than 30–40% compared to untreated D4-Lnn OGD/R cells. However, against the background of this suppression of necrosis, there was no significant increase in the percentage of viable cells. After exposure to 10 and 30 μg/mL D4-Lnn, a more pronounced decrease in the number of cells with necrosis was observed, while when using a concentration of 100 μg/mL D4-Lnn, the number of necrotic cells increased sharply (Figure 2C). Interestingly, low concentrations of D4-Lnn (1 and 3µg/mL) promoted the development of the early stages of apoptosis in cells, and the number of such cells increased by approximately 50% compared to OGD/R cells. Treatment with 10, 30, and 100 µg/mL D4-Lnn did not significantly increase the number of cells with early apoptosis. When evaluating cells characterized by the presence of late stages of apoptosis, it was found that all investigated concentrations of D4-Lnn led to a decrease in such cells. The most pronounced protective effect against the toxic action of OGD/R was characteristic for 10µg/mL D4-Lnn. The number of cells with apoptotic signs decreased by almost four times compared to OGD/R cells. Interestingly, 10 µg/mL of non-deuterated Linoleic acid (Figure 2B) did not have a similar cytoprotective effect in OGD/R. In cortical culture was found in the early and late stages of apoptosis in 14 ± 8% and 33 ± 12% of cells, and 60 ± 19% necrotic cells (Figure 2C—yellow bars; Supplementary, Figure S2).

Thus, exogenous D4-Lnn dose-dependently suppressed OGD/R-induced necrosis of cerebral cortex cells, and concentrations of 10 µg/mL D4-Lnn and higher lead to inhibition of apoptosis.

### 2.2. The Neuroprotective Effect of D4-Lnn Occurs Due to the Suppression of Ca^2+^ Signals in Cerebral Cortex Cells in Response to Ischemia-like Conditions and Depends on the Incubation Time

**Figure 3:**
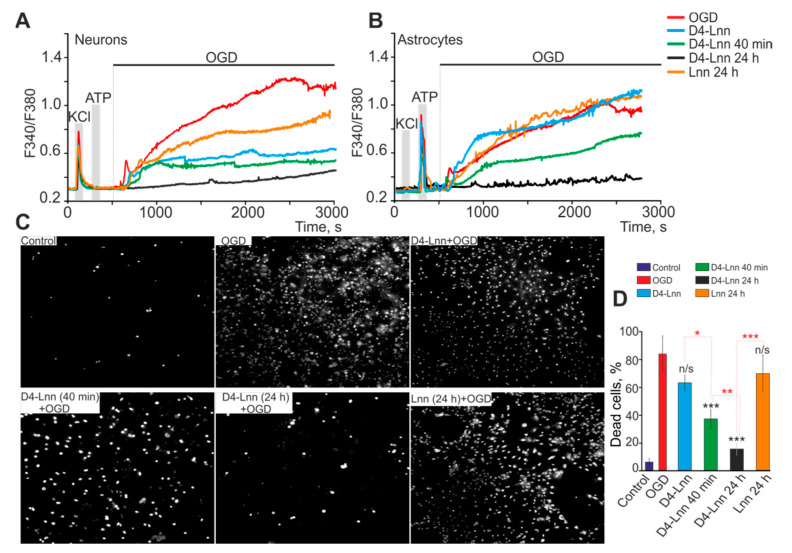
Protective effect of 10 µg/mL D4-Lnn on mouse cortical cells: (**A**,**B**) Ca^2+^ -signals of neurons (**A**) and astrocytes (**B**) during a 40 min OGD (red traces), 40 min OGD with D4-Lnn (in media, blue traces), after 40 min D4-Lnn (green traces), and 24 h preincubation (black traces) with D4-Lnn or after 24 h pre-incubation with Lnn (orange traces). The figure shows the averaged Ca^2+^ signals obtained from several tens of cells for each curve. Individual Ca^2+^ responses of neurons and astrocytes are presented in Supplementary, Figure S3. (**C**) Images of cortical cell culture in Propidium Iodide fluorescence detection channel before the experiment (Control) and after 40-min OGD or OGD with different time treatment with 10 µg/mL D4-Lnn. The white dots represent the nuclei of necrotic cells. For panels A and B, the averaged Ca^2+^ signals in one experiment, obtained from several tens of astrocytes and neurons, are presented. Panel C presents typical vitality test results corresponding to experiments from panels A and C. (**D**) The percentage of PI^+^ cortical cells that died due to OGD-induced necrosis in the control (without OGD), in the absence of D4-Lnn (OGD) and after different time incubation with 10 µg/mL D4-Lnn or Lnn (24 h preincubation). Statistical significance was assessed using one-way ANOVA followed by the Tukey–Kramer test. Comparison of experimental groups relative to OGD: n/s—data not significant (*p* > 0.05), * *p* < 0.05, ** *p* < 0.01, and *** *p* < 0.001. Statistical differences based on incubation time with D4-Lnn are indicated by red asterisks. The results obtained on 4 cell cultures are presented. N (number of animals used for cell cultures preparation) = 4.

It is known that, with pathological changes in the body, as a rule, significant disturbances in the mechanisms of calcium signaling occur, leading to corresponding changes in the functions of nerve cells. Ischemia also belongs to such pathological states of the nervous system; therefore, changes in calcium signaling in neuroglial cells before and after treatment of cells with D4-Lnn under conditions of ischemia were very important. In response to OGD (40 min), a biphasic increase in [Ca^2+^]_i_ was recorded in neurons and astrocytes (Figure 3A,B—red curves), and the phase of the global increase in [Ca^2+^]_i_ correlated with necrotic death (Figure 3C—OGD) 82 ± 18% of cells (Figure 3D). Application of 10 µg/mL D4-Lnn to cortical cells during OGD did not affect the first reversible phase of the OGD-induced increase in [Ca^2+^]_i_, but in neurons (Figure 3A—blue curve) the global growth phase was significantly suppressed, which did not occur in astrocytes (Figure 3B—blue curve). Under OGD conditions and application of 10 µg/mL D4-Lnn, a decrease in the number of necrotic cells to 63 ± 15% was observed (Figure 3C—D4-Lnn + OGD; Figure 3D), probably due to the survival of neuronal populations. Interestingly, the application of 10 µg/mL D4-Lnn causes the generation of Ca^2+^ pulses or Ca^2+^ oscillations exclusively in astrocytes (Figure 3B—blue curve; Supplementary, Figure S3D). As our previous studies have shown, the cytoprotective effect of substances can increase with an increase in incubation time [13,14,15]. Pre-incubation of the cells with 10 µg/mL D4-Lnn for 40 min resulted in the inhibition of the reversible phase of the OGD-induced increase in [Ca^2+^]_i_ and an even more pronounced suppression of the global increase in [Ca^2+^]_i_ in neurons (Figure 3A—green curve). In astrocytes, a 40-min pre-incubation with 10 μg/mL D4-Lnn decreased the amplitude of the first phase of the Ca^2+^ signal in response to OGD and caused suppression of the global increase in [Ca^2+^]_i_ (Figure 3B—green curve), but to a lesser extent compared with neurons. At the same time, cell death also decreased and amounted to 38 ± 9% (Figure 3C,D). Cultivation of the cerebral cortex cells with 10 μg/mL D4-Lnn for 24 h resulted in the complete suppression of both phases of the OGD-induced increase in [Ca^2+^]_i_ in neurons (Figure 3A) and astrocytes (Figure 3B), which correlated with a decrease in the number necrotic cells up to 18 ± 5% (Figure 3C,D). At the same time, Ca^2+^ oscillations were observed in astrocytes under OGD conditions, arising after a lag period after the creation of ischemic conditions (Supplementary, Figure S3H). Incubation of the studied cells with 10 µg/mL Lnn for 24 h was less effective in suppressing the OGD-induced global increase in [Ca^2+^]_i_ in neurons (Figure 3A—orange curve) compared to the D4-Lnn. Ca^2+^ -signals in astrocytes did not significantly differ in shape and amplitude after incubation of studied cells with Lnn compared to untreated cells (Figure 3B—orange curve). Cell death as a result of an OGD-induced global increase in [Ca^2+^]_i_ after 24 h treatment with Lnn was 72 ± 21% (Figure 3C,D).

Thus, the cytoprotective effect of D4-Lnn depended on the incubation time and after 24 h led to complete inhibition of OGD-induced Ca^2+^ signals in neurons and astrocytes of the cerebral cortex, which was accompanied by the suppression of necrotic cell death. A fast protective effect of D4-Lnn was established for neurons when suppression of the global increase in [Ca^2+^]_i_ was observed both in the presence of deuterated fatty acid and after 40 min of preincubation.

### 2.3. D4-Lnn Suppresses OGD-Induced ROS Production and Alters the Expression Profile of Genes Encoding Redox Proteins

**Figure 4:**
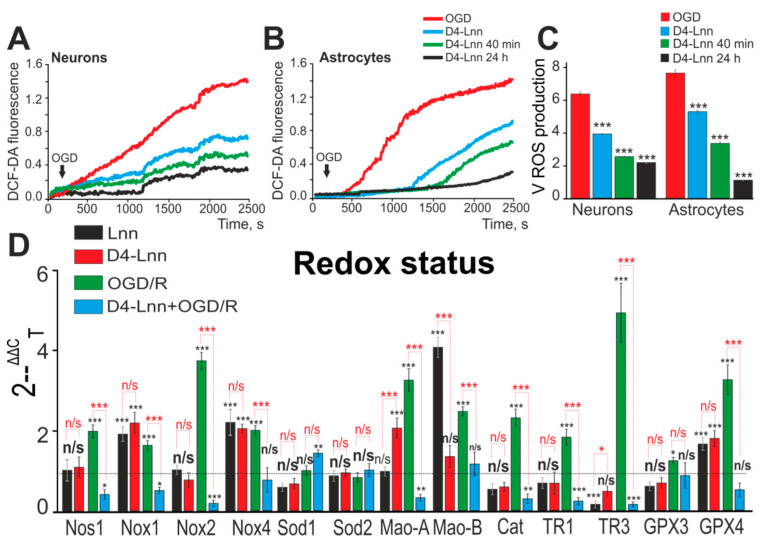
ROS production in neurons (**A**) and astrocytes (**B**) under OGD conditions versus incubation time with 10 µg/mL D4-Lnn. Average kinetics of ROS production obtained from several tens of neurons and astrocytes for each curve are presented in one experiment. (**C**) The rate of ROS formation in neurons and astrocytes during OGD, depending on the time treatment with D4-Lnn. Average values obtained from three separate cell cultures are presented here. (**D**) Effect of 24 h preincubation with 10 µg/mL D4-Lnn or non-deuterated Lnn (Lnn) on baseline and OGD/R-induced expression level of genes encoding proteins regulating cell redox status. Gene expression in control cells are marked by dashed line. Statistical significance was assessed using one-way ANOVA followed by the Tukey–Kramer test. Comparison of experimental groups regarding control: n/s—data not significant (*p* > 0.05), * *p* < 0.05, ** *p* < 0.01 and *** *p* < 0.001. Comparison of experimental groups relative to each other is indicated in red. For panels D and E, the number of RNA samples is 5. N (number of animals used for cell cultures preparation) = 5.

It is known that, during OGD, there is an increase in the generation of ROS in neurons and astrocytes, and this process is most pronounced in the cytosol of astrocytes, in comparison with neurons [7]. To detect ROS production predominantly in the cytosol, cortical cells were loaded with a DCF-DA probe. In response to OGD, there was a rapid increase in the rate of ROS production in neurons (Figure 4A—red curve), while in astrocytes, ROS production was observed after a lag period (3 ± 1 min) (Figure 4B—red curve). Under OGD conditions and with 10 µg/mL D4-Lnn application, the rate of ROS production in neurons was suppressed by 38% (Figure 4A—blue curve; Figure 4C) and by 27% in astrocytes, and an increase in the duration of the lag phase, preceding ROS production was observed (Figure 4B—blue curve; Figure 4C). An increase in the incubation time with D4-Lnn to 40 min and 24 h resulted in a suppression of the rate of ROS production in neurons by 56% and 59%, respectively (Figure 4A,C). In astrocytes, there was an increase in the duration of the lag period preceding OGD-induced ROS production in direct proportion to the time of incubation with D4-Lnn (Figure 4B). Whereas, after the preincubation of cells with D4-Lnn during 40 min and 24 h, suppression of the rate of ROS production by 53% and 79.4%, respectively, was also observed (Figure 4C).

Using real-time PCR, it was found that 24 h treatment of cell cultures with 10 µg/mL D4-Lnn led to a change in the basic expression of several genes encoding proteins regulating redox-status. There was an increase in the expression of Nox1, Nox4, Mao-A, and GPX4 by 2.3, 2.2, 2.2, and 1.9 times, respectively, while the TR3 level decreased by 49% (Figure 4D—red bars). Whereas treatment with Lnn led to an increase in the basic expression of Nox1, Nox4, Mao -B, and GPX4 by 1.9, 2.4., 4.2, 1.7 times (Figure 4D—black bars).

24 h after OGD/R, a significant increase in the expression of genes Nos1, Nox1, Nox2, Nox4, Mao-A, Mao-B by 2, 1.6, 3.8, 2.1, 3.2, 2.6 times was observed, respectively (Figure 4D—green bars), which could adversely affect the antioxidant properties of cells. At the same time, there was a compensatory increase in the expression level of genes Cat (Catalase), TR1, TR3, GPX3 and GPX4 by 2.2, 1.8, 4.7, 1.3 and 3.3 times, respectively (Figure 4D—green bars). Preincubation of cells with 10 µg/mL D4-Lnn leads to inhibition of the expression of Nos1, Nox1, Nox2 and Mao-A relative to OGD/R by 70%, 67%, 93% and 64%, respectively (Figure 4D—blue bars).

Thus, D4-Lnn leads to rapid suppression of OGD-induced ROS formation in the cytosol of neurons and this effect persisted after treatment of cells during 24 h with 10 µg/mL D4-Lnn, while in astrocytes this effect increased in proportion to the incubation time with D4-Lnn, including the lag phase was forming. Deuterated and non-deuterated linoleic acid affects the basic expression pattern of the same genes that regulate cell redox-status. And D4-Lnn effectively suppressed OGD/R-induced expression of genes encoding proteins involved in the regulation of ROS production and their toxic effects.

### 2.4. Incubation of Cortical Cells with D4-Lnn Influenced the Basal and OGD/R-Mediated Expression Levels of Genes Encoding Proteins Responsible for the Induction of Apoptosis, Inflammation, and Excitotoxicity

**Figure 5:**
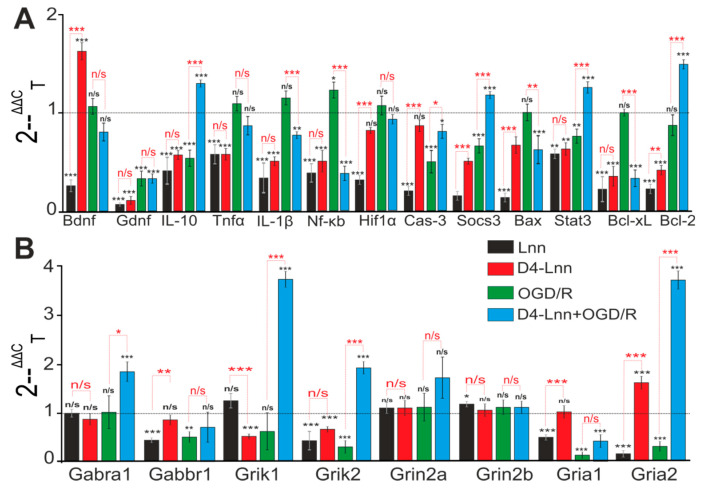
Effect of 24 h incubation of cells with 10 µM D4-Lnn on baseline and OGD/R induced levels of expression of genes encoding proteins regulating apoptosis, inflammatory status (**A**) and receptors (**B**). Gene expression in control cells are marked by dashed line. Statistical significance was assessed using one-way ANOVA followed by the Tukey-Kramer test. Comparison of experimental groups regarding control:n/s–data not significant (*p* > 0.05), * *p* < 0.05, ** *p* < 0.01 and *** *p* < 0.001. Comparison of experimental groups relative to each other is indicated in red. The number of RNA samples is 5. N (number of animals used for cell cultures preparation) = 5.

Incubation of cortical cells for 24 h with 10 µg/mL Lnn or D4-Lnn was accompanied by a change in the baseline expression level of genes encoding key proteins responsible for the normal functioning of neuronal and glial cells. Lnn caused a decrease in the basic expression of 12 of the 13 studied genes, brain neurotrophic factor BDNF and GDNF, anti-inflammatory cytokine IL-10, transcription factor Hif1α, antiapoptotic genes, Socs3, Stat3, Bcl-2. Such a change in expression may indicate a proapoptotic effect of Lnn (Figure 5A—black bars).

On the other hand, suppression of the basic expression of proinflammatory factors—Tnfα, IL-1β, Nf-κB, and proapoptotic genes—Bax and Bcl-xL can be regarded as an anti-apoptotic effect. D4-Lnn had a similar effect on the basic genes expression, however a decrease in the mRNA expression level of the Hif1α gene was not observed. The expression of BDNF mRNA was almost doubled compared to the control, which, under hypoxia/ischemia conditions, may be key for the protection of neuroglial networks (Figure 5A, red bars). Inhibition of the expression of three genes-encoding neuroprotective proteins IL-10, Socs3, and Bcl-2, was observed after OGD/R, while the level of mRNA expression of the proapoptotic genes Nf-κB and Cas-3, on the contrary, increased (Figure 5A—green columns). Pre-incubation of cortical cells with D4-Lnn for 24 h changed the OGD/R-induced gene expression profile—there was an increase in the expression of protective genes (IL-10, Socs3, Stat3, Bcl-2) and, at the same time, a suppression of proapoptotic and proinflammatory genes Il -1β, Nf-κB, Cas-3, Bax, and Bcl-xL (Figure 5A—blue bars). A decrease in the expression levels of genes encoding the subunits of ionotropic receptors Gabbr1, Grik1, Gria1, and Gria2 (Figure 5B—green bars) was also observed under OGD/R conditions in the cerebral cortex culture, which may indicate an impaired neurotransmission and the induction of hyperexcitation of the neural network. Pre-incubation with D4-Lnn led to the opposite effect: an increase in the expression of the genes Gabra1, Grik1 and Grik2, and Gria2 was observed (Figure 5B—blue bars).

### 2.5. D4-Lnn Caused the Generation of Ca^2+^ Signals in Astrocytes of the Cerebral Cortex through the Activation of the Phosphoinositide Signaling Pathway

**Figure 6:**
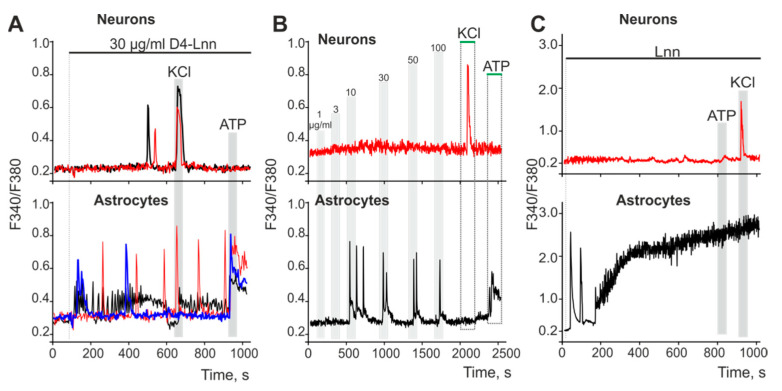
Application of D4-Lnn or Lnn induces the generation of Ca^2+^ signals in astrocytes, but not in neurons of the cerebral cortex: (**A**) Application of 30 µg/mL D4-Lnn causes the rapid generation of three types of Ca^2+^ signals in astrocytes, and Ca^2+^ responses occur in neurons (single, no more than 0.5% of the total population of neurons) after a lag period. Individual Ca^2+^ responses are presented. (**B**) Short-term applications (30 s) of increasing concentrations of D4-Lnn to cell cultures of the cerebral cortex. After washing with D4-Lnn, the registration of the [Ca^2+^]_i_ dynamics was suspended for 10 min. The averaged curves obtained from several tens of cells are presented. (**C**) Application of 30 µg/mL non-deuterated Lnn causes the rapid generation of biphasic Ca^2+^ signals in astrocytes and does not cause Ca^2+^ responses in neurons. The averaged curves obtained from several tens of cells are presented. Number of parallel coverslips with cell cultures in each analysis = 5. N (number of animals used for cell cultures preparation) = 3.

An addition of 10 µg/mL D4-Lnn to cell culture of brain cortex prior to induction of ischemia caused the generation of Ca^2+^ signals in astrocytes but not neurons (Figure 6B, blue curve; Supplementary, Figure S3C,D). Figure 6A shows that the application of 30 µg/mL D4-Lnn caused the rapid generation of three types of Ca^2+^ signals in astrocytes: transient signals (blue curve), high-amplitude low-frequency (red curve), and low-amplitude high-frequency (black curve) Ca^2+^ oscillations. In some neurons (Figure 6A—top), single Ca^2+^ pulses could be recorded, occurring 8–10 min after the activation of astrocytes. Such Ca^2+^ signals in neurons occurred due to the activation of neurons by astrocytes. To determine the dependence of the activation of Ca^2+^ signals in the cells of the cerebral cortex on the concentration of D4-Lnn (Figure 6B), short-term (30 s) applications of D4-Lnn were performed. After washing from D4-Lnn, the experiment was paused for 10 min to restore the Ca^2+^-transporting systems of the cells. On average, astrocytes began to respond with the generation of Ca^2+^ signals to the application of 10 µg/mL of D4-Lnn, and an increase in the concentration of D4-Lnn did not cause an increase in the amplitude of the Ca^2+^ signal (Figure 6B—bottom). In neurons, none of the D4-Lnn concentrations elicited noticeable Ca^2+^ signals (Figure 6B—top). The non-deuterated form Lnn has significant differences. The application of 30 µg/mL Lnn did not affect the Ca^2+^ dynamics of neurons (Figure 6C—red curve), but caused the generation of the same type of Ca^2+^ signals in astrocytes (Figure 6C—black curve), characterized by the presence of two phases. The first phase of the Lnn Ca^2+^ signals induced by Lnn consisted of several Ca^2+^ impulses, followed by a global increase in [Ca^2+^] i, and the absence of Ca^2+^ signals from astrocytes for the application of ATP may indicate a violation of the Ca^2+^ transporting systems of cells.

**Figure 7:**
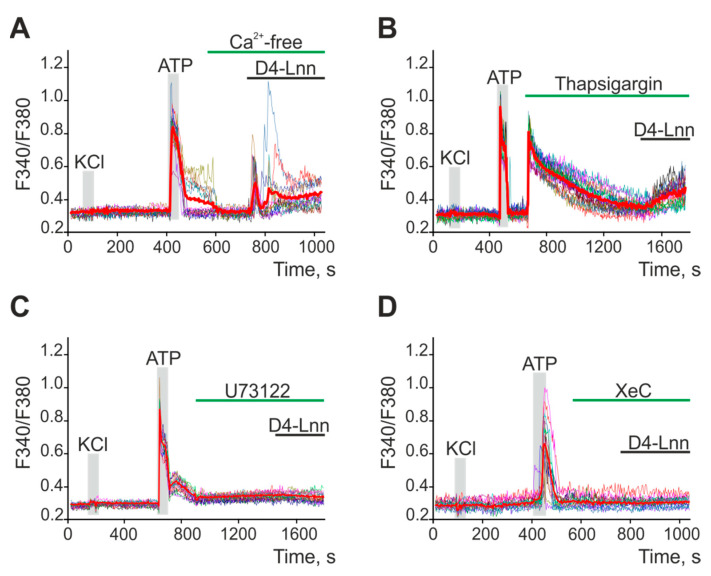
Application of D4-Lnn induces the generation of Ca^2+^ signals in astrocytes of the cerebral cortex through the phosphoinositide signaling cascade. (**A**,**B**) Addition of 30 µg/mL D4-Lnn in a calcium-free medium containing 0.5 mM EGTA (**A**) and after emptying the ER with 10 µM thapsigargin in a calcium-free medium with EGTA (**B**). (**C**,**D**) Application 30 µg/mL D4-Lnn against the background of PLC blockers (**C**, U73122, 5 µM) and IP_3_R (**D**, XeC, XestospongineC, 1 µM). The figure shows the Ca^2+^ signals of individual astrocytes and their mean value (red curves). Number of parallel coverslips with cell cultures in each analysis = 4. N (number of animals used for cell cultures preparation) = 3.

Since neurons did not respond to D4-Lnn application, experiments on establishing the signaling pathway responsible for the increase in [Ca^2+^]_i_ in astrocytes are presented below. To generate Ca^2+^ responses, cells use the entrance of Ca^2+^ ions from outside or its mobilization from intracellular ER stores. The application of 30 µg/mL D4-Lnn in a calcium-free medium led to the generation of Ca^2+^ signals in astrocytes (Figure 7A). However, emptying the ER with thapsigargin (Figure 7B) inhibited the generation of Ca^2+^ astrocyte responses to D4-Lnn application. Inhibition of key proteins of the phosphoinositide signaling pathway, PLC (Figure 7C) and IP_3_R (Figure 7D), also prevented the generation of Ca^2+^ responses in astrocytes.

Thus, the generation of transient Ca^2+^ signals by astrocytes in response to exogenous D4-Lnn occurred due to PLC activation, IP_3_ synthesis, and mobilization of Ca^2+^ ions from the ER via IP_3_R. At the same time, non-deuterated Lnn also activates the Ca^2+^ signaling system of astrocytes, but leads to disruption of calcium homeostasis of cells and a global increase in [Ca^2+^]_i_, which is a symptom of cell death.

### 2.6. D4-Lnn Resulted in Astrocyte Reactivation

**Figure 8:**
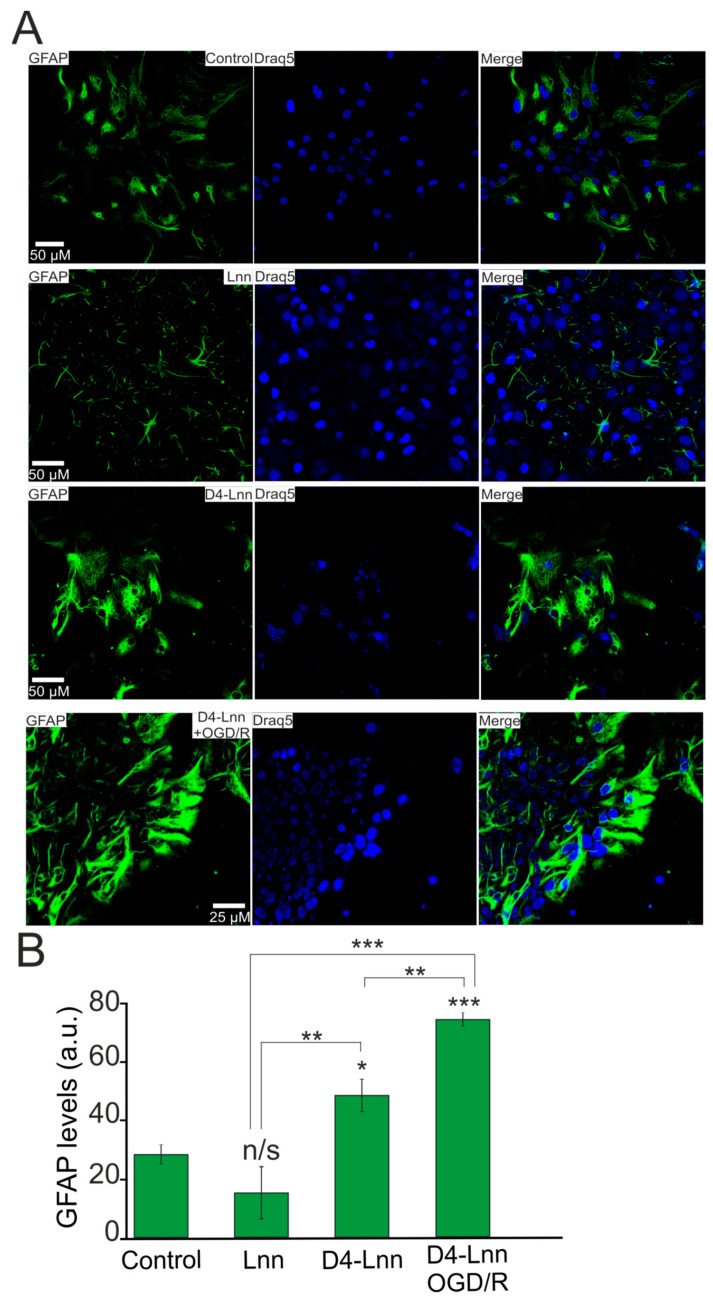
Pre-incubation of cortical cells with 10 µM D4-Lnn for 24 h causes an increase in GFAP protein expression. The reactivation effect persisted 24 h after OGD/R: (**A**) Immunostaining cortical cells with antibodies against GFAP in the control, after 24-h pre-incubation with 10 µg/mL D4-Lnn, 10 µg/mL Lnn, and after 40 min OGD with 24-h reoxygenation (cells 24-h pre-incubated with 10 µg/mL D4-Lnn before OGD/R). Draq5—nuclei staining. (**B**) Intensity levels of GFAP were determined by confocal imaging. We analyzed individual cells which had fluorescence of secondary antibodies. The quantitative data reflecting the level of GFAP expression are presented as fluorescence intensity values in summary bar charts (mean +/− SEM). The values were averaged by 150 cells for each column. The results obtained after immunostaining agree well with the data of fluorescent presented in panels (**A**). Each value is the mean ± SE (*n* ≥ 3, *p* < 0.05). Statistical significance was assessed using one-way ANOVA followed by the Tukey–Kramer test. n/s—data not significant (*p* > 0.05), * *p* < 0.05, ** *p* < 0.01, *** *p* < 0.001. For repeats, 4 separate cell cultures were used. N (number of animals used for cell cultures preparation) = 4.

Activation of the Ca^2+^ signaling system is associated with the process of reactivation of astrocytes, which is capable of triggering protective signaling pathways in astrocytes themselves, as well as in the neural network, due to the secretion of gliotransmitters [16]. After a 24 h incubation of cortical cells with 10 μg/mL D4-Lnn, the fluorescence of antibodies against GFAP, which is a known marker of astrocyte activation and reactivity, was increased by 84% relative to control (Figure 8A,B). Under OGD/R conditions and with the application of 10 µg/mL D4-Lnn, the GFAP protein level increased by 63% compared with the experimental group D4-Lnn and almost three times compared with the control group (Figure 8A,B—D4-Lnn + OGD/R). At the same time, pre-incubation with 10 µg/mL of non-deuterated Lnn did not significantly affect the GFAP level (Figure 8A,B—Lnn).

Thus, 10 μg/mL D4-Lnn caused an increase in the expression of the GFAP protein in astrocytes, and this effect of astrocyte reactivation was enhanced after OGD/R.

## 3. Discussion

PUFAs are substrates for oxidation reactions in cells and act as modulators of various biochemical reactions, signaling cascades, etc. If the metabolism of PUFAs is impaired, their excessive accumulation in tissues can occur in the body and, as a result, can lead to the development of fatty infiltration of the liver and pancreas (steatosis), fatty degeneration of the heart, and impaired neurotransmission in the brain [17,18]. With ischemia and post-ischemic reperfusion in the affected tissue, the concentration of PUFAs increases to 30–40 µM, and the risk of death is increased manifold in patients with obesity, metabolic syndrome, and type II diabetes [19].

Linoleic and α-linoleic acids are essential fatty acids and precursors of long-chain PUFAs and must be obtained from the diet, as mammals cannot synthesize them de novo. In our experiments, 24-h incubation of cortical cells with Lnn did not reduce cell death after OGD/R, and only in neurons, but not in astrocytes, was the global increase in [Ca^2+^]_i_ during OGD somewhat suppressed, which correlated with a change in the gene expression profile, coding proteins responsible for the activation of damaging intracellular signaling pathways. There is evidence of the neuroprotective effect of Lnn in models of hypoxic-ischemic neuronal injury [20,21]. It was shown that Lnn promoted neuronal stem cell survival under conditions of NMDA-induced excitotoxicity [22]. In the kainate model of epilepsy, only intranasal treatment with Lnn, unlike other PUFAs, resulted in the suppression of cell death in the CA1 and CA3 regions of the hippocampus [23] through the activation of NF-κB [24,25], which, in addition to activation of the inflammatory response, is involved in the mechanism of neuroplasticity. On the other hand, there is strong evidence for the damaging effect of Lnn through non-metabolic conversion to “oxidized linoleic acid metabolites” (OXLAMs) during the autoxidation of Lnn or by enzymes—lipoxygenase (LOX), cyclooxygenase (COX), cytochromeP450 (CYP450), and soluble epoxidehydrolase [26,27]. The concentration of OXLAMs increases in the cerebral cortex and cerebellum when animals are kept on a diet high in Lnn [28]. During ischemia, there is also an increase in OXLAMs in different parts of the brain, which leads to tissue damage [29]. It was found that the prenatal intake of diets high in the ratio of Lnn to α-Lnn was associated with a two-fold increase in the risk of delayed psychomotor and mental development in 6-month infants [30]. Our experiments provide some insight into the effect of Lnn and D4-Lnn on the patterns of basic and OGD/R-induced expression of genes encoding signaling proteins and ionotropic receptor subunits. The more stable D4-Lnn is less prone to oxidation and the formation of OXLAMs, causing mainly antiapoptotic and anti-inflammatory effects on the cerebral cortex cells.

According to our data, the presence of D4-Lnn in the ischemic medium led to a significant inhibition of the Ca^2+^ signal amplitude in neurons, and preliminary incubation with D4-Lnn for 24 h completely suppressed the global increase in [Ca^2+^]_i_ under OGD conditions. In astrocytes, the effect of D4-Lnn on OGD-induced Ca^2+^ dynamics was recorded after 40 min and 24 h of pre-incubation, which is also well known for the anti-inflammatory cytokine IL-10, antioxidants Prx-6, taxifolin, and the neurotrophic factor BDNF [2,7,13,30,31]. PUFAs can suppress neuronal death through the inhibition of glutamatergic neurotransmission. Lnn injections, unlike palmitic acid, suppress kainate-induced epileptiform activity [23]. PUFAs can potentially suppress an excessive increase in [Ca^2+^]_i_ through the inhibition of EPSP and glutamatergic transmission, acting on some voltage-sensitive K^+^ channels [32], inhibiting glutamate transporters [33] and regulating the activity of NMDA receptors [34]. There is evidence for the ability of PUFAs to partially inhibit voltage-sensitive Na^+^ channels [35] and voltage-sensitive Na^+^ channels [36]. As our experiments showed, Lnn and D4-Lnn altered the basic and OGD/R-induced expression of genes encoding subunits of excitatory kainate and AMPA receptors, as well as inhibitory GABA-B receptors, without affecting NMDA- and GABA-A- receptors. However, D4-Lnn led to a change in the expression pattern of receptor subunits, which could affect the inhibition of Ca^2+^ entry into cells during OGD/R. This effect was not observed when using Lnn.

During hypoxia, ischemia, and reoxygenation, there is a powerful increase in lipid peroxidation due to the production of ROS [8,14]. Autooxidation of PUFAs is a chain reaction activated by an increase in ROS concentration. As a result of this process, the toxic products malondialdehyde, 4-hydroxy-2-nonenal, acromine, and α, β-unsaturated carbonyls are formed. In the brain, during various pathological processes that activate lipid peroxidation, a toxic product—4-hydroxy-2-nonenal—is the cause of necrotic neuronal death. In addition, hydrophilic regions appear in the hydrophilic layer of the membrane due to an increase in the content of fatty acid hydroperoxides, and, as water and Ca^2+^ ions begin to penetrate the cell, the fluidity of the cell membrane changes, and membrane proteins gain increased mobility in the bilayer [37]. At the cellular level, this destabilization of lipid metabolism causes swelling of cells, organelles, and their destruction. Pre-incubation of cell cultures with D4-Lnn led to a decrease in the rate of ROS formation in neurons and especially in astrocytes, which correlated with the suppression of OGD/R-induced changes in the expression of genes encoding proteins of the redox status of cells. It is known that the level of NOS expression is enhanced by OGD/R, however our experiments showed that pre-incubation with D4-Lnn suppressed NOS expression and oxidative stress in general. It is known that the level of NOS expression increases during inflammatory processes in Parkinson’s disease, which leads to an increase in NO production, protein nitrosylation, and the induction of apoptosis [38,39].

**Figure 9:**
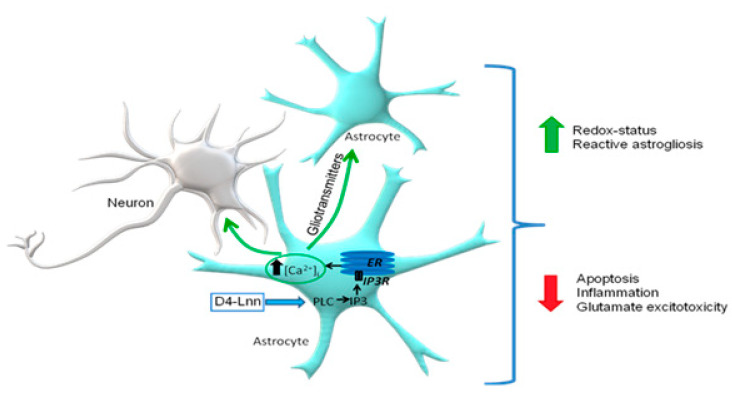
The putative scheme of activation of the Ca^2+^ signaling system of cortical astrocytes and the cytoprotective mechanism by exogenous D4-Lnn.

During the OGD/R period, a significant part of ROS is formed in brain cells after NADPH oxidases (NOX) activation. NOX are bound to the cell membrane and generate superoxide into the extracellular space, which is converted into secondary ROS by enzymatic and non-enzymatic pathways [40]. In our experiments, 24 h treatment of cortical cells with D4-Lnn led to a decrease in the expression levels of genes encoding NOX1 and NOX2, which are, among other things, regulators of proinflammatory signaling pathways involving TNFα [41]. This may indicate a protective effect of D4-Lnn at the level of regulation of these proteins. It is known that NOX2 is mainly expressed in astrocytes, while NOX4 (whose expression did not depend on D4-Lnn), on the contrary, is expressed in neurons [42]. This effect on NOX2 may determine the astrocyte-specific effect of D4-Lnn on ROS production during OGD. Monoamine oxidases (MAO) are also a source of ROS in brain cells [43], and their blockers inhibit the formation of dopamine-induced mPTP pores [44]. As our experiments showed, D4-Lnn suppresses the expression of MAO-A, which can also contribute to cytoprotection under OGD/R conditions. Known enzymes with antioxidant properties are catalases and thioredoxin reductases [45,46], the expression of which increased under OGD/R conditions. This was especially characteristic of TR3, the expression level of which increased by almost five times compared to the control. Treatment of cells with D4-Lnn promoted a significant decrease in the expression of catalase, TR1, and 3, which may indicate the absence of the need to activate these enzymes under conditions of a reduced level of oxidative stress in cells. Of interest is the selective action of D4-Lnn on the Ca^2+^ signaling system of astrocytes, causing the activation of the phosphoinositide signaling system and the mobilization of Ca^2+^ ions from the ER via IP3R (Figure 9). It is known that exogenous PUFAs are freely transported across the plasma membrane of cells through the passive diffusion mechanism, the “flip-flop” process [47], and with the help of carrier proteins [48]. Likewise, deuterated FA also readily penetrate tissues and cells. FA, through the modulation of the Ca^2+^ signaling system and the expression of intracellular receptors ryanodine and IP_3_R, can partially restore signaling deficiency in white adipocytes obtained from obese mice [49]. It is known that PUFAs can lead to an increase in ROS production, TLR4, or ER activation [50,51,52]. One of the PUFAs signaling action mechanisms is an increase in the concentration of their metabolite, diacylglycerol (DAG), formed as a result of PLC activity, which can activate PKC, IKK-β, JNK, and other kinases [52,53,54,55].

The activation of Ca^2+^ signals in response to the addition of D4-Lnn probably caused the reactivity of astrocytes, which was confirmed in our experiments by staining cortical cultures with antibodies against GFAP. It is known that the aggregation of α-synuclein, which occurs in Parkinson’s disease, stimulates the release of proinflammatory cytokines by reactive astrocytes, which contributes to neuronal damage [56]. It has been shown that this is accompanied by an increase in NOS expression and NO production, as well as general oxidative stress in neurons and glial cells [57]. At the same time, microglial activation decreased after D-PUFA treatment, which suppressed the inflammatory response in the rat brain with a viral construct carrying the α-synuclein sequence [9]. It is known that neuroinflammation and ischemia can be caused by two different types of reactive astrocytes (“A1” and “A2”) [58]. A1 astrocytes largely activate many of the genes in the classical complement cascade that have previously been shown to be destructive to synapses. In contrast, A2 astrocytes are characterized by a defense mechanism that manifests itself in the activation of many neurotrophic factors. Reactive astrocytes during ischemia exhibit a molecular phenotype that suggests that they may be beneficial or protective; however, despite well-established common features, reactive astrocyte gliosis is very heterogeneous. In our experiments, the Ca^2+^ signaling system of astrocytes was activated in response to the addition of D4-Lnn, which correlated with the reactivation of astrocytes and the suppression of necrotic neuronal death after OGD/R.

## 4. Materials and Methods

### 4.1. Ethical Approval

Experimental protocols were approved by the Bioethics Committee of the Institute of Cell Biophysics. Experiments were carried out according to Act708n (23 August 2010) of the Russian Federation National Ministry of Public Health, which states the rules of laboratory practice for the care and use of laboratory animals, and the Council Directive 2010/63 EU of the European Parliament on the protection of animals used for scientific purposes.

### 4.2. Reagents

From Sigma–Aldrich, St. Louis, USA: Adenosine 5′-triphosphate disodium salt hydrate (ATP, A1852), Hanks′ Balanced Salt Solution (HBSS, H4641), HEPES sodium salt (H7006), Xestospongine C (X2628), U73122 (U6756), Thapsigargin (T9033); from Molecular probes, Eugene, Oregon, USA: Hoechst 33342 (H1399), Propidium iodide (P1304MP), Linoleic Acid (CAS 60-33-3); from Evrogene, Moscow, Russia: MMLV reverse transcriptase (SK022S), SYBR Green I PCR Master Mix (PK147L), Primers; from Thermo Fisher Scientific, Waltham, Massachusetts, USA: Fura-2AM (Cat. #F1221), H_2_DCF-DA (Cat. #D399), B-27 Supplement (Cat. # 17504044), Neurobasal-A Medium (Cat. 10888022); Purified mouse monoclonal anti-GFAP antibody (BioLegend, RRID: AB_2632644); Donkey polyclonal secondary antibody to mouse IgG—H&L (Alexa Fluor-594) (Abcam, RRID: AB_2732073); Linoleic Acid-d4 (Santa Cruz Biotechnology, CAS 79050-23-0).

### 4.3. Cortical Cell Culture Preparation

Cell cultures were prepared as described in detail previously [59]. Briefly, 0–1 day mice (NMRI mouse line) were euthanized and decapitated. The extracted cortex was washed with Mg^2+^- and Ca^2+^-free Versene solution and minced with scissors. Then, the tissue fragments were digested with 1% trypsin solution for 10 min at 37 °C and washed two times with a cold Neurobasal-A medium. Trypsinized tissue was gently triturated with a pipette, and the debris was then carefully removed with a pipette tip. The obtained cell suspension was seeded on polyethyleneimine-coated glass coverslips and grown for 10 days in vitro in the cell culture medium composed of Neurobasal-A medium, supplement B-27 (2%), and 0.5 mM glutamine. On average, a suspension with a cell concentration of 2.5 ± 1.5 million/mL was obtained from a piece of the mouse cerebral cortex. A 250 ± 50 thousand cells were placed on each coverslips. On the 10 th day of in vitro cultivation, 100 ± 50 thousand cells were found on the coverslips, since, when the cells were isolated using trypsin, 30–40% of the cells in suspension did not survive in the first days of cultivation. Experiments were carried out on the 10th day of cultivation. The neuronal cells and astrocytes were maintained together in cell culture. The ratio of neurons to astrocytes in the used cell cultures, revealed by staining with antibodies against NeuN (neuronal nucleic marker), averaged 33 ± 16% of astrocytes and 67 ± 11% of neurons (Supplementary, Figure S4).

The drugs and D4-Lnn were added into the culture medium under sterile conditions in the case of experiments with 24 h pre-incubation. Then, the cell cultures were washed after the pre-incubation with Hank’s balanced salt solution and used in experiments.

### 4.4. The Technique for Simulation of Ischemia-like Conditions

Ischemia-like conditions (oxygen–glucose deprivation, OGD) were simulated by omitting glucose (HBSS medium without glucose) and the displacement of dissolved oxygen with argon in the leak-proof system [7]. Oxygen tensions reached values 30–40 mm Hg or less within 20 min after the beginning of displacement. OGD conditions lasting for 40 min were created by supplying the OGD-medium into the chamber with cultured cortical cells. Constant argon feed into the experimental chamber was used to prevent the contact of the OGD-medium with the atmospheric air.

### 4.5. Assessment of Cell Viability and Apoptosis

Hoechst 33342 (2 µM) and propidium iodide (1 µM) were used to evaluate the number of dead cells in the cell cultures before and after OGD. The cells were stained for 5 min with the probes diluted in HBSS and then rinsed with HBSS. Fluorescence of the probes was detected with an inverted fluorescent microscope Zeiss Axio Observer Z1 using Filter Set 01 and Filter Set 20. Discrimination of early and late apoptotic cells was performed according to the previously described method [13,60]. Five different areas of each cell culture were analyzed.

To distinguish neurons and astrocytes, we used short-term applications of 35 mM KCl and 10 µM ATP before the main experiments. This method was described in detail in our previous work [14]. Briefly, KCl induces depolarization of excitable cells, which contain a wide range of voltage-gated cation channels. KCl-induced depolarization promotes the opening of voltage-gated calcium channels in neurons (predominantly L-type channels). The conductivity and density of cation channels in astrocytes are insufficient to evoke high-amplitude Ca^2+^-response to KCl application.

### 4.6. Extraction of RNA

Mag Jet RNA Kit (Thermo Fisher Scientific, Waltham, MA, USA) was used for the extraction of total RNA. The RNA quality was estimated by electrophoresis in the presence of 1 μg/mL ethidium bromide (2% agarose gel in Tris/Borate/EDTA buffer). The concentration of the extracted RNA was determined with a NanoDrop 1000c spectrophotometer. RevertAid H Minus First Strand cDNA Synthesis Kit (Thermo Fisher Scientific, Waltham, MA, USA) was used for reverse transcription of total RNA.

### 4.7. Real-Time Polymerase Chain Reaction (RT-qPCR)

Each PCR was performed in a 25 μL mixture composed of 5 μL of qPCRmix-HS SYBR (Evrogen, Moscow, Russia), 1 μL (0.2 μM) of the primer mix, 17 μL water (RNase-free), 1 μL cDNA. Dtlite 5 Real-Time PCR System (DNA-technology, Moscow, Russia) was used for amplification. The amplification process consisted of the initial 5 min denaturation at 95 °C, 40 cycles of 30 s denaturation at 95 °C, 20 s annealing at 60–62 °C, and 20 s extension step at 72 °C. The final extension was performed for 10 min at 72 °C. All sequences of the used primers were designed with FAST PCR 5.4 and NCBI Primer-BLAST software. The data were analyzed with Dtlite 5 software (DNA-technology, Moscow, Russia). The expression of the studied genes was normalized to gene encoding Glyceraldehyde 3-phosphate dehydrogenase (GAPDH). Data were analyzed using Livak’s method [61].

### 4.8. Immunocytochemical Method

To detect GFAP in cells, we used an immunocytochemical assay. The cells were fixed with 4% paraformaldehyde +0.25% glutaraldehyde in PBS for 20 min and washed three times with ice-cold PBS for 5 min. Glutaraldehyde was added into the fixative solution to minimize the washing of antibodies from cells during permeabilization. To permeabilize cells, we used 0.1% Triton X-100 solution for 15 min. Fixed cells were incubated in 10% donkey serum for 30 min at room temperature to block non-specific antibody binding sites. The cells were then incubated with primary antibodies against investigated proteins for 12 h at 4 °C. The fixed cells were subsequently washed with PBS (3 times for 5 min) and probed with secondary antibodies conjugated with a fluorescent label. We used purified mouse monoclonal anti-GFAP antibody (BioLegend, RRID: AB_2632644) and donkey polyclonal secondary antibody to mouse IgG—H&L (Alexa Fluor-594) (Abcam, RRID: AB_2732073). Dilutions of primary and secondary antibodies were performed according to the manufacturer’s recommendations for immunocytochemical staining. The fluorescence of antibodies was visualized with an inverted confocal microscope Leica TCS SP5 (Leica, Germany). Registration of the secondary antibodies’ fluorescence for the control and experimental groups of cell cultures was carried out at the same microscope setting. Fluorescence analysis was performed in ImageJ 2002 software (RRID: SCR_003070) using the Analyze particles and time-series analyzer plugins.

### 4.9. Fluorescent Ca^2+^ Measurements

To measure the changes in [Ca^2+^]_i_, cell cultures were stained with Fura-2 (4 µM; 40 min incubation; 37 °C) in Hank’s balanced salt solution (HBSS) composed of (mM): 156 NaCl, 3 KCl, 2 MgSO_4_, 1.25 KH_2_PO_4_, 2 CaCl_2_, 10 glucose, balanced with 10 HEPES, pH 7.4. To measure [Ca^2+^]_i_, we used inverted motorized microscope Leica DMI6000B with a high-speed monochrome CCD-camera HAMAMATSU C9100. For excitation and registration of Fura-2 fluorescence, we used the FU-2 filter set (Leica, Wetzlar, Germany) with excitation filters BP340/30 and BP387/15, beam splitter FT-410, and emission filter BP510/84. Illuminator Leica EL6000 with a high-pressure mercury lamp was used as a source of excitation light.

### 4.10. Fluorescent ROS Measurements

For ROS production detection, cortical cell cultures were stained with H_2_DCF-DA (10 µM, 20 min incubation; 37 °C) and washed three times before the experiments. To measure the ROS production, we used the inverted motorized microscope Leica DMI6000B with a high-speed monochrome CCD-camera HAMAMATSU C9100 and a high-speed light filter replacing system Leica’s Ultra-Fast Filter. For excitation of DCFH_2_-DA, we used the L5 filter set (Leica, Germany) with excitation filter BP480/40, dichroic mirror 505, and emission filter 527/30. We determined the shape of ROS production rates under oxygen-glucose deprivation (OGD).

### 4.11. Statistical Analysis

All presented data were obtained from three cell cultures. All values are given as mean ± standard error (SEM) or as individual signals. One-way analysis of variance (ANOVA) followed by the Tukey–Kramer post-hoc test was used for multiple group comparisons. MS Excel, ImageJ, Origin 2016 (OriginLab, Northampton, MA, USA), and Prism GraphPad 7 (GraphPad Software, RRID: SCR_002798) software was used for data and statistical analysis.

## 5. Conclusions

It was shown that deuteration of polyunsaturated linoleic fatty acid significantly enhances its protective properties against oxidative stress caused by ischemia-like conditions, followed by reoxygenation (OGD/R) in the cells of the mouse cerebral cortex in vitro (Figure 9). It was found that D4-Lnn completely inhibits necrosis and significantly reduces apoptotic cell death in proportion to an increase in its concentration in the medium, cause’s reactivation of astrocytes by activating the phosphoinositide calcium system, and regulates the expression of a large number of genes encoding enzymes that are involved in the induction of apoptosis, necrosis, inflammation, and excitotoxicity.

## Data Availability

The data presented in this study are available on request from the corresponding author.

## Supplementary Materials

The following are available online at https://www.mdpi.com/article/10.3390/ijms222413216/s1.
