# Supplementary material for: The Protective Mechanism of Deuterated Linoleic Acid Involves the Activation of the Ca2+ Signaling System of Astrocytes in Ischemia In Vitro
Source: Int J Mol Sci. 2021 Dec 8;22(24):13216. doi: 10.3390/ijms222413216 (PMC8706680; doi:10.3390/ijms222413216)
Supplement: Supplementary file 1 [file ijms-22-13216-s001.zip › ijms-1468950-supplementary.pdf]

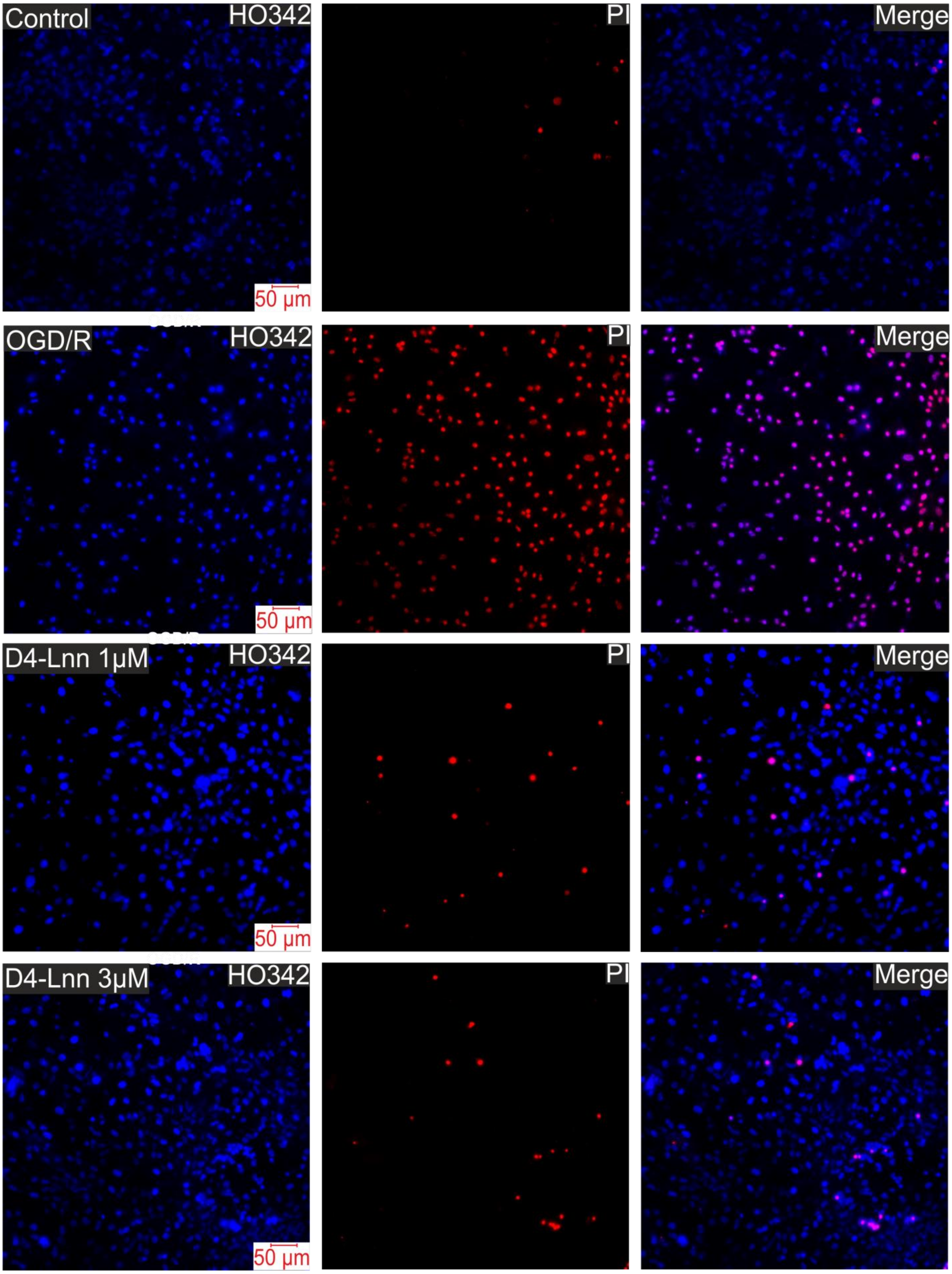

SUPPLEMENTARY, FIGURE S1. Induction of necrosis and apoptosis in cerebral cortex cells 24 h after OGD/R, depending on the preliminary incubation with 1 and 3 µg D4-Lnn.
Double staining of cells with Hoechst 33342 (HO342), Propidium iodide (PI) and merge (Merge) between Hoechst342 and PI in control (no exposure), 24 h after OGD/R (OGD/R) and 24 h after OGD/R depending on the preliminary (24 h) incubation of cells with 1 and 3 µg D4-Lnn.The images shown in the figure correspond to the data in figure 1 of the text of the article.

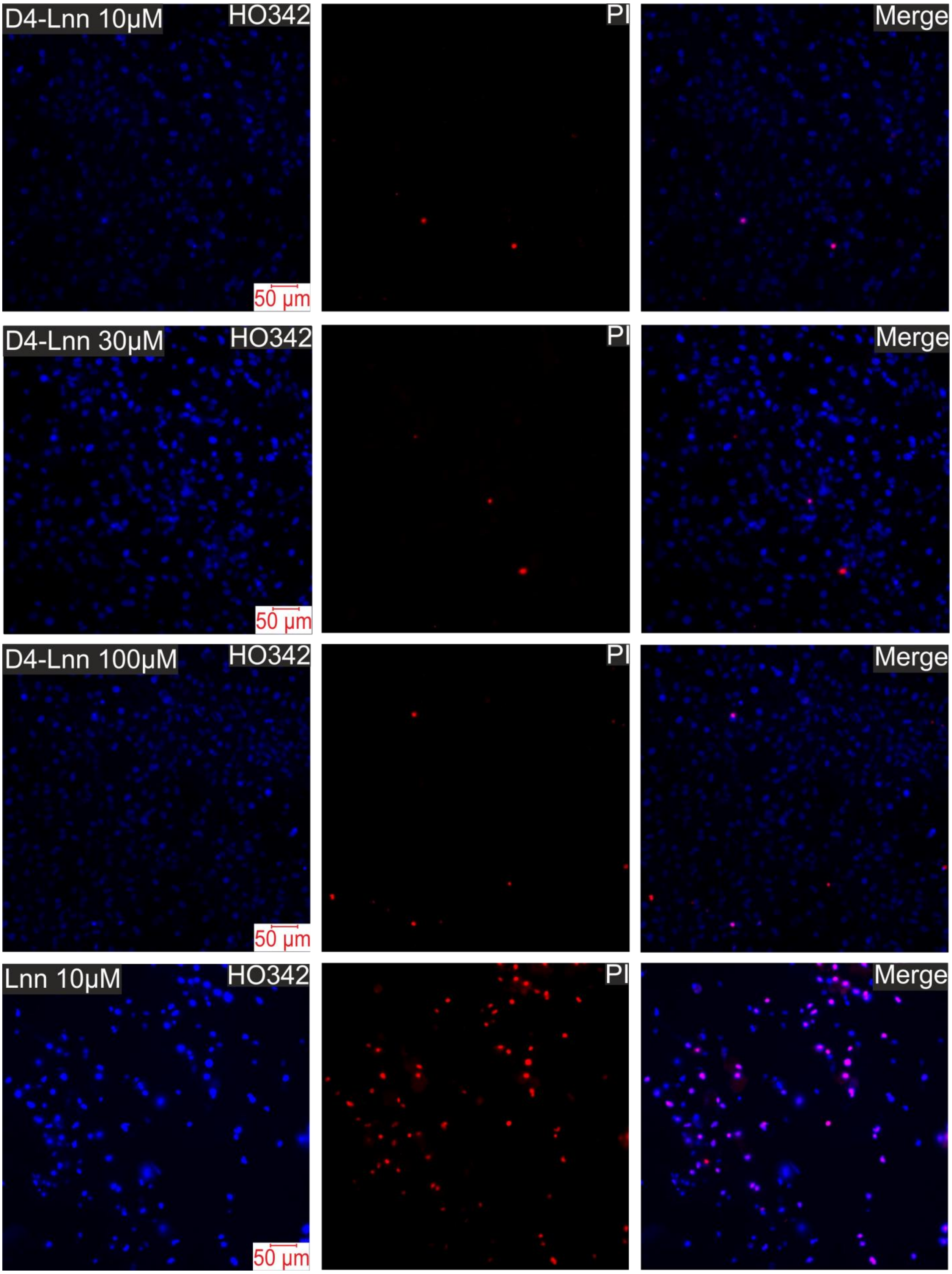

SUPPLEMENTARY, FIGURE S2. Induction of necrosis and apoptosis in cerebral cortex cells 24 h after OGD/R, depending on the preliminary incubation with 10, 30 100 µg D4-Lnn or 10 µg Lin.

Double staining of cells with Hoechst 33342 (HO342), Propidium iodide (PI) and merge (Merge) between Hoechst342 and PI depending on the preliminary (24 h) incubation of cells with D4-Lnn or Lnn. The images shown in the figure correspond to the data in fig.1 of the article.

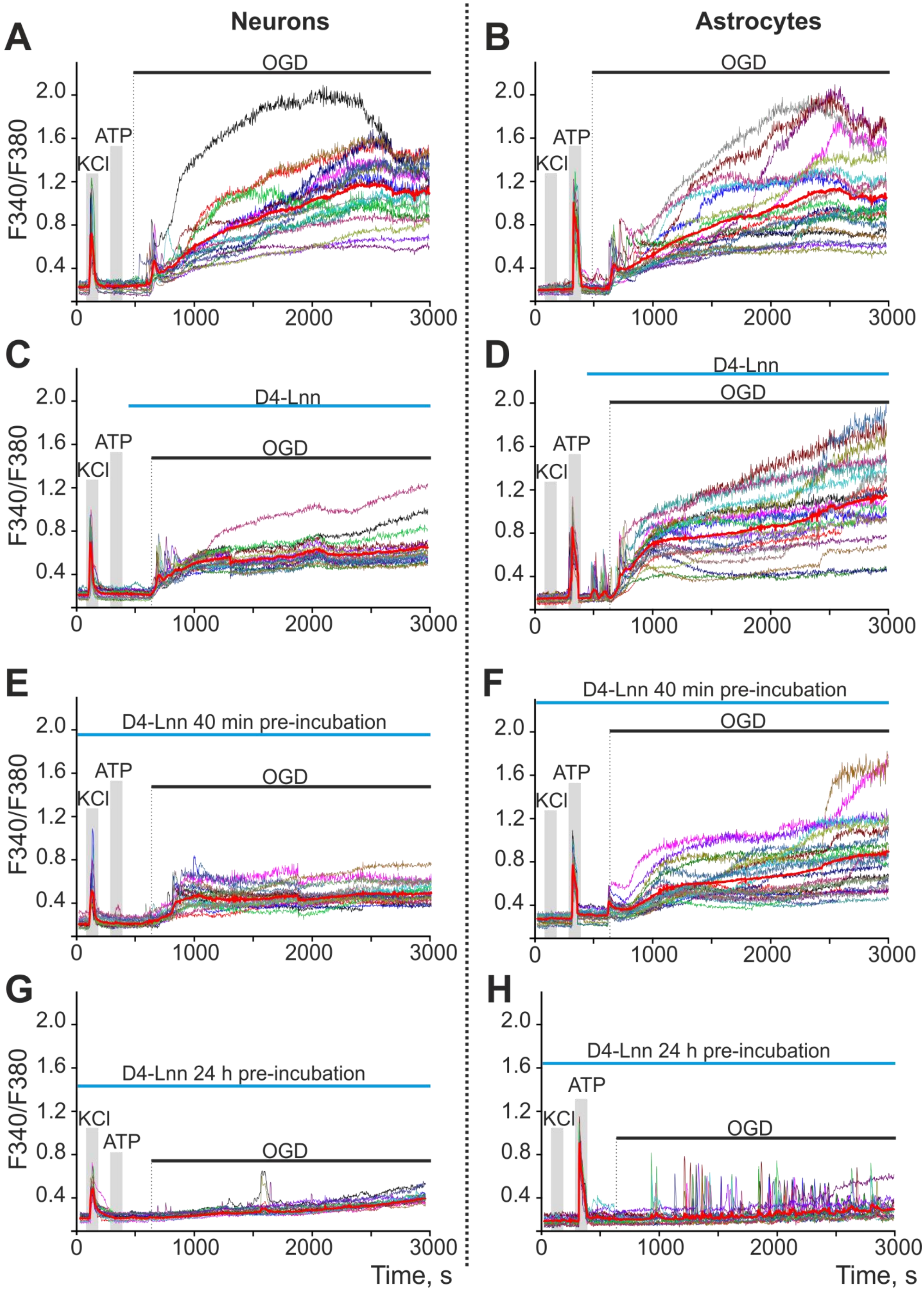

SUPPLEMENTARY, FIGURE S3. Effect of different incubation times of cortical cultures with 10 μg D4-Lnn on OGD-induced Ca^{2+} signals in neurons and astrocytes.

The figure shows the Ca^{2+} signals of the cells and their averaged values (red curves).

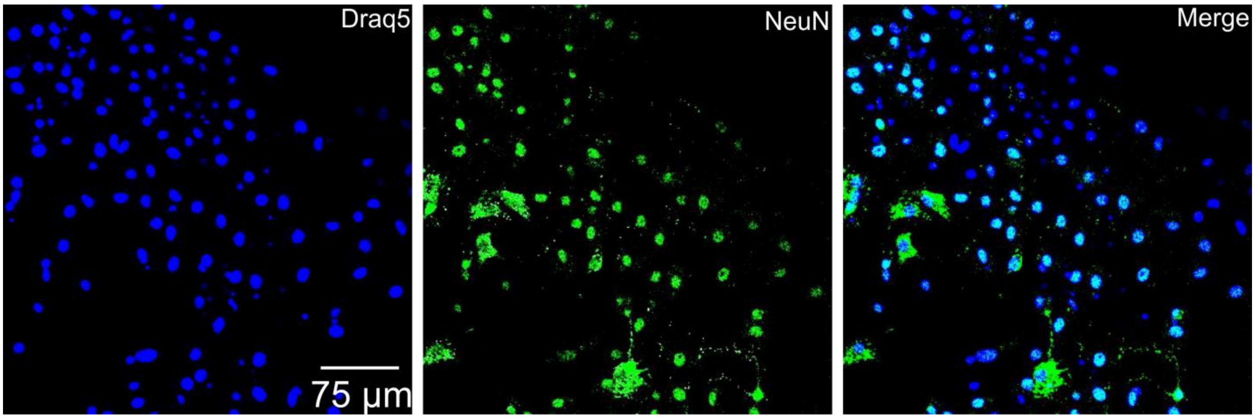

SUPPLEMENTARY, FIGURE S4. Immunocytochemical staining of cortical cell culture with neuronal marker, antibodies against NeuN. The nuclei of all cells stained with Draq5 are shown in blue.
